# Enhanced surveillance to assess the presence of Sindbis and Batai virus in mosquito populations at an urban zoo in the United Kingdom

**DOI:** 10.1186/s13071-025-07149-4

**Published:** 2025-12-07

**Authors:** Madhujot Jagdev, Insiyah Parekh, Robert C. Bruce, Simon Spiro, Colin J. Johnston, Anthony J. Abbott, Ethan Wrigglesworth, Paul Pearce-Kelly, Alexander G. C. Vaux, Jolyon M. Medlock, Nicholas Johnson, Arran J. Folly, Mirjam Schilling

**Affiliations:** 1 Virology Department, Animal and Plant Health Agency, Woodham Lane, Addlestone, Surrey KT15 3NB UK; 2 Zoological Society of London, Regent’s Park, London, NW1 4RY UK; 3 Medical Entomology and Zoonoses Ecology Group, UK Health Security Agency, Salisbury, SP4 0JG UK

**Keywords:** *Culex pipiens*, *Culex torrentium*, Usutu virus, West Nile virus, Arbovirus, Mosquito-borne disease

## Abstract

**Background:**

Sindbis virus (SINV) and Batai virus (BATV) are emerging zoonotic arboviruses with a growing number of detections in Europe. Recent SINV outbreaks in northern Europe and high BATV seroprevalence in sheep, goats, and cattle in Germany emphasise the threat they pose to both animal and human health. Despite their presence in countries of similar latitude and climate, neither of these viruses have been detected in the UK.

**Methods:**

Zoos are strategic sentinel sites for disease surveillance because they are well monitored and possess a high diversity of animal species. Located in southeast England, where the climate and vector prevalence may provide suitable conditions for viral emergence, London Zoo was selected as the sampling site for SINV and BATV prevalence in mosquito samples between September 2022 and January 2024. In 2020, it was also the first location where Usutu virus was detected in the UK. Adult mosquitoes were collected during host-seeking and overwintering seasons while larvae were collected in the summer months.

**Results:**

A total of 8477 mosquito specimens were analysed, representing all mosquito stages, i.e. including host-seeking and overwintering mosquitoes as well as adults that had emerged from larvae. Mosquitoes of the *Culex pipiens*/*Culex torrentium* complex were the most abundant, accounting for 97.5% of the total. Molecular analysis using quantitative polymerase chain reaction was performed to test for SINV and BATV; however, none of the samples tested positive.

**Conclusions:**

These results suggest that neither SINV nor BATV actively circulated in the sampled area during the study period. The findings provide baseline data for arbovirus surveillance in the UK, particularly at London Zoo, which is home to diverse bird populations that might be potential sentinel populations for viral emergence. Future studies that obtain molecular and serological data on birds or mammals housed at the zoo would complement our surveillance efforts on the emergence or prevalence of SINV and BATV in the UK. This study focused on a single location, but extending sampling and monitoring to other sites across the UK, especially in the southeast, is crucial to strengthening the UK’s preparedness and response strategies in case SINV and BATV emerge in the country in the future.

**Supplementary Information:**

The online version contains supplementary material available at 10.1186/s13071-025-07149-4.

## Background

Climatic changes, the vector competence of indigenous mosquito species, as well as the repeated introduction and geographic expansion of exotic mosquito species, have together increased the risk of emerging vector-borne diseases in Europe. In recent years, the number of confirmed cases of zoonotic arboviruses, such as Sindbis virus (SINV) and Batai virus (BATV), has increased, making them an emerging threat to human as well as animal health [1].

Sindbis virus, a mosquito-borne pathogen belonging to the genus* Alphavirus* (family Togaviridae), is one of the most widely distributed arboviruses globally, with occurrences documented in Australia, Africa, Asia, and Europe [2, 3]. Mosquitoes from the genera *Culex*, *Culiseta*, and *Aedes* are the primary vectors for transmitting the virus to humans and other animals during active host-seeking. The birds they take blood meals from, particularly those from the orders Passeriformes and Anseriformes, are assumed to serve as key reservoir hosts and therefore play a crucial role in sustaining the enzootic transmission cycle of SINV and may facilitate its broader geographic spread through migration [4]. Additionally, a warming climate causing enhanced vector activity increases the risk for virus transmission from mosquitoes to humans and other animals [5]. In humans, SINV infection can lead to Sindbis fever, which is characterized by fever, rash, and arthritis [1]. Although asymptomatic infections are not uncommon, symptoms can persist for years [6]. Signs of disease in animals are rarely reported. However, neurological disease has been observed in horses in Africa [7].

In contrast to SINV, Batai virus (BATV), in the genus* Orthobunyavirus*, has been less extensively studied yet is widespread across Asia and Africa, with an increasing number of detections in several European countries [8]. Although it has thus far only been reported to cause relatively mild clinical symptoms, such as febrile illness, in humans, it should be considered a potential public health risk due to its particle stability and long-lasting infectivity, the risk of recombination of its segmented genome, as well as its known co-circulation with other arboviruses [9, 10]. In livestock BATV, co-infections or reassortment have been reported in cases of abortion, premature birth, and congenital defects [11–13]. Similar to SINV, transmission of this enveloped, single-stranded, negative-sense (RNA) virus occurs predominantly via species of mosquito genera such as *Anopheles*, *Culex*, and *Ochlerotatus* [14], which are highly abundant across Europe.

Mosquito species known to transmit both SINV and BATV are not only widely distributed across mainland Europe but are also native to the UK [14, 15]. An incursion of either SINV or BATV may impact the health of both humans and other animals in the UK. Despite this risk, only a few studies have investigated the prevalence of these viruses in the UK. Low levels of neutralizing antibodies against SINV were found in UK-resident and migratory birds [16] and in the serum of a small number of sentinel chickens from Cambridgeshire in 2004 [17], indicating that the chickens may have been exposed to SINV, or at least to a very similar virus that then caused a cross-reactive immune response. However, neither BATV nor SINV has been detected in the UK to date [18].

The detection of neutralizing antibodies against SINV in the UK, along with the prevalence of the virus in other European countries and the proven vector competence of native mosquitoes, suggest a risk for both animals and humans in the UK [19–21]. SINV outbreaks are frequently recorded in Scandinavian countries [22, 23], and seroprevalence data indicate that livestock in several European countries are regularly exposed to BATV, with cattle, sheep, and swine being the most likely natural reservoirs [8, 24–26]. Additionally, migratory birds are believed to play a significant role in the broad geographic distribution of BATV in Europe and Asia [13], which increases the risk of new virus introductions into the UK. A recent study investigating the introduction of SINV to Sweden suggests that even a single introduction into a new geographical area can lead to SINV establishment and spread [27]. Phylogenetic analysis suggests that SINV was introduced into Sweden only once, in the 1920s, from which point it spread eastward and southward in two independent events during the 1960s and 1970s.

These findings underscore the need for improved surveillance in the UK, as highlighted by the detection of Usutu virus (USUV), the UK’s first case of a mosquito-borne viral zoonosis [28], in a blackbird in 2020, and recently also West Nile virus (WNV) in mosquitoes [29]. This is especially important in the southeast, where the risk of incursion is known to be particularly high [30]. This area has not only been shown to be permissive for novel incursions, but also for viral establishment [31]. Only a clearer insight into the prevalence of potential arboviral threats in specific locations and species will help us to direct the control of mosquito populations to limit virus transmission.

The aim of the present study was to address this knowledge gap through an enhanced surveillance approach, by collecting potential vectors for SINV and BATV between 2022 and 2024 and investigating virus prevalence in three different phases of a mosquito’s life: host-seeking, overwintering and, indirectly, at the larval stage. As zoos are well monitored, they are ideal sentinel locations for surveillance activities. The additional diversity of both wild native and exotic species on site that might function as hosts further enhances the potential to detect new incursions early in zoos. Therefore, we focused our enhanced surveillance efforts on London Zoo, the site where USUV was detected in 2020 as the UK’s first mosquito-borne viral zoonosis [28].

## Methods

### Mosquito trapping

**Fig. 1:**
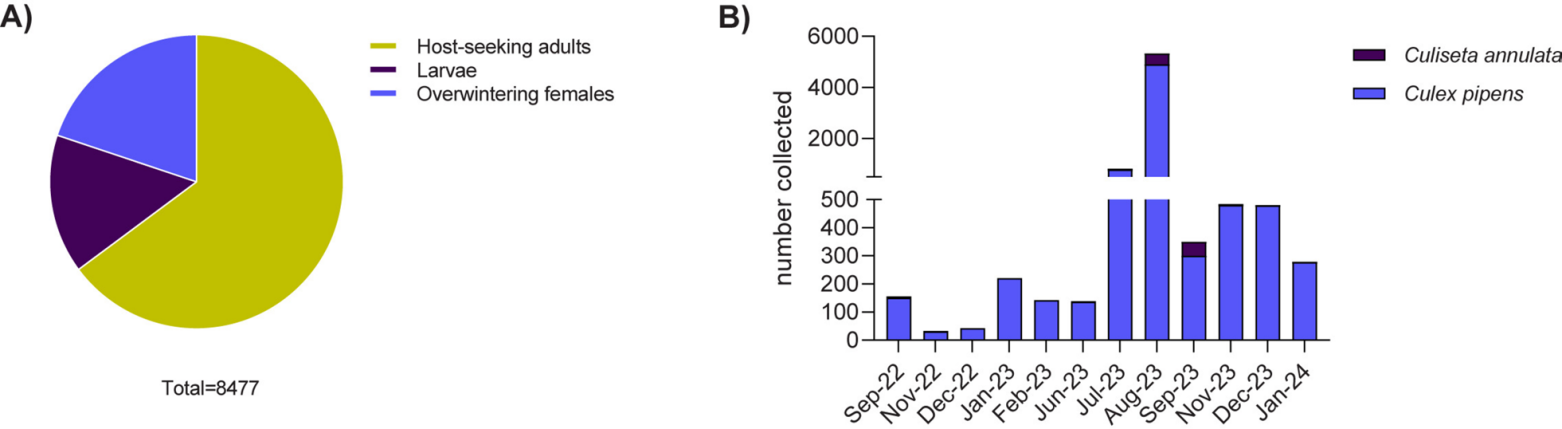
Proportional representation of stages and species of mosquitoes collected. **A** Pie chart illustrating the percentage of mosquitoes collected, categorized into three groups—host-seeking mosquitoes, larvae, and overwintering mosquitoes—relative to the total mosquito population sampled. **B** Monthly collection of mosquitoes by species and total counts

Mosquitoes were trapped between 2022 and 2024. As the dynamics of mosquito populations, and thereby arboviral circulation within mosquito communities, are influenced by a combination of temporal, environmental, and ecological factors, we attempted to take snapshots of virus prevalence in different mosquito stages, including adult host-seeking mosquitoes, larvae, and overwintering females, at different times of the year. Sampling periods were chosen based on weather conditions likely to support arbovirus circulation or overwintering (Fig. 1). Adult host-seeking mosquitoes were captured using CO_2_-baited BG-Sentinel traps (Biogents, Regensburg, Germany) following the first detection of USUV in birds on site during the summer season. Locations for trapping were distributed across the zoo, strategically targeting areas with open water pools, such as the Penguin Beach or African Bird Safari (Supplementary Table 1). Both locations had been shown to house bird species seropositive for USUV and these might therefore act as good sentinel species for mosquito-borne disease surveillance [31]. A handheld vacuum-powered aspirator (Bug Buster, Katcha®) was used to collect resting overwintering adults, while larvae were collected from various water sources at London Zoo using Pasteur pipettes. Additionally, we set up and monitored water-filled pots as traps for larvae in strategic locations (Supplementary Table 1). Locations for larvae collection were chosen based on previous USUV detections as well as enclosures where animals had been detected as seropositive for flaviviruses or USUV specifically, despite the absence of disease [31]. Larvae were collected as first, second and third instars. Hibernacula for the collection of overwintering mosquitoes were identified by London Zoo staff where resting females had been observed during the winter months (Supplementary Table 1).

The collected overwintering mosquitoes were transported to the Animal and Plant Health Agency insectary, where they were reared under controlled conditions, as follows: 25 °C temperature, 50% relative humidity, and a 12:12-h light–dark cycle (following protocols established by Schilling et al. [32]). Rearing continued for 3–4 weeks, during which the mosquitoes were monitored daily. The aim was to end the dormancy and increase the metabolic activity of the mosquitoes as this might reactivate viral replication and facilitate viral detection by quantitative polymerase chain reaction (qPCR). A similar approach used to obtain virus sequences from overwintering *Culex pipiens* had been successful for WNV [33] and St. Louis encephalitis virus [34] in two independent studies undertaken in the USA. Any dead mosquitoes were recorded and stored at −80 °C to preserve them for subsequent molecular analysis.

To additionally monitor potential arbovirus transmission in and throughout the larval stage, first-, second- and third-instar larvae were collected from standing water pools and oviposition traps (darkened plastic containers filled with a mix of fresh water and water from larval habitats) in summer 2023 using Pasteur pipettes and aquatic nets following protocols established by Schilling et al. [32]. Collected larvae were transferred to a new larval tray containing fresh water and reared to adult mosquitoes under the aforementioned conditions. Both male and female mosquitoes were frozen at −80 °C 3 weeks after emergence before molecular analysis.

### Morphological identification of mosquitoes

Prior to freezing, mosquitoes were identified morphologically to the lowest taxonomic certainty following the taxonomic keys outlined by Snow [35]. Females of *Culex pipiens* (biotypes *Culex pipiens pipiens* and *Culex pipiens molestus*) and *Culex torrentium* cannot be differentiated by morphological characteristics [36]. Recent studies indicate the presence of all three taxa in Greater London, with *Culex pipiens* sensu stricto being the most prevalent [37]. Our collection therefore likely contained *Cx. pipiens* and *Cx. torrentium*.

### Nucleic acid extraction

RNA extraction from mosquitoes was conducted following the protocol outlined in the RNeasy Kit (catalogue no. 74106; QIAGEN, Manchester, UK). Mosquitoes were homogenised in pools of 10 in 350 µl of Buffer RLT with 5-mm stainless steel beads (catalogue no. 69989; QIAGEN) on a TissueLyser II (QIAGEN). RNA was eluted in 40 µl of RNase-free water.

### Viral testing of mosquitoes

The screening for SINV and BATV RNA was performed using a TaqMan-based real time–qPCR (RT-qPCR) QuantiTect Probe RT-PCR Kit (catalogue no. 204443; QIAGEN) assay and a SYBR Green-based RT-qPCR assay (catalogue no. 1725150; iTaq^™^ Universal SYBR^®^ Green One-Step Kit; Bio-Rad), respectively.

For SINV detection, primers and probe were designed to target a 134-nucleotide region within the non-structural protein 1 of the SINV reference strain Edsbyn, as described by Jöst et al. [38]: forward primer (5′–CACWCCAAATGACCATGC–3′), reverse primer (5′–KGTGCTCGGAAWACATTC–3′), and probe (5′–FAM-CAGAGCATTTTCGCATCTGGC-BHQ-1–3′). The thermal cycling conditions consisted of complementary DNA synthesis at 48 °C for 30 min, reverse transcriptase inactivation at 95 °C for 10 min, followed by 45 cycles of denaturation at 95 °C for 15 s and annealing/extension at 55 °C for 1 min. Fluorescence data were collected during the annealing/extension phase of each cycle. SINV (strain 5.3; Germany) RNA served as the positive control [38].

For BATV detection, primers were designed to amplify a 200-nucleotide region of the L segment of the BATV genome, as described by Jöst et al. [39]: forward primer (5′–GATGGCGATTTCCTGATTAT–3′) and reverse primer (5′–TGACCCCAAGAGTTTCCTATTAT–3′). The thermal cycling conditions comprised reverse transcription at 50 °C for 10 min, reverse transcriptase inactivation at 95 °C for 5 min, followed by 40 cycles of denaturation at 95 °C for 10 s and annealing/extension at 55 °C for 30 s. Fluorescence data were acquired during the annealing/extension step. BATV (strain 53.2) RNA (Germany) served as the positive control [39].

### Detection threshold estimation

To evaluate the sensitivity of our sampling approach, we calculated the minimum detectable prevalence based on the total number of host-seeking female mosquitoes tested (*n* = 5496). Assuming that there was one positive sample, the minimum observed prevalence was determined by dividing 1 by the total sample size and multiplying by 100 to express the result as a percentage.

## Results

A total of 8477 specimens were collected over the study period, from August 2022 to January 2024, inclusive, across nine sites at London Zoo. Of the total mosquitoes collected, 5496 were host-seeking mosquitoes, 1297 had emerged from larvae, and 1684 were overwintering mosquitoes (Fig. 1A). The majority of the collected mosquitoes were morphologically identified as *Culex pipiens/Culex torrentium* (93.9%), with a smaller number identified as *Culiseta annulata* (6.1%). Monthly collections varied, with the highest number of host-seeking mosquitoes collected in August 2023 (*n* = 4990) and the lowest number of overwintering females in November 2022 (*n* = 33) (Fig. 1B). The estimated detection threshold for SINV and BATV, based on our sample size of host-seeking females (*n* = 5496), corresponds to a minimum observed prevalence of 0.0182%.

**Fig. 2:**
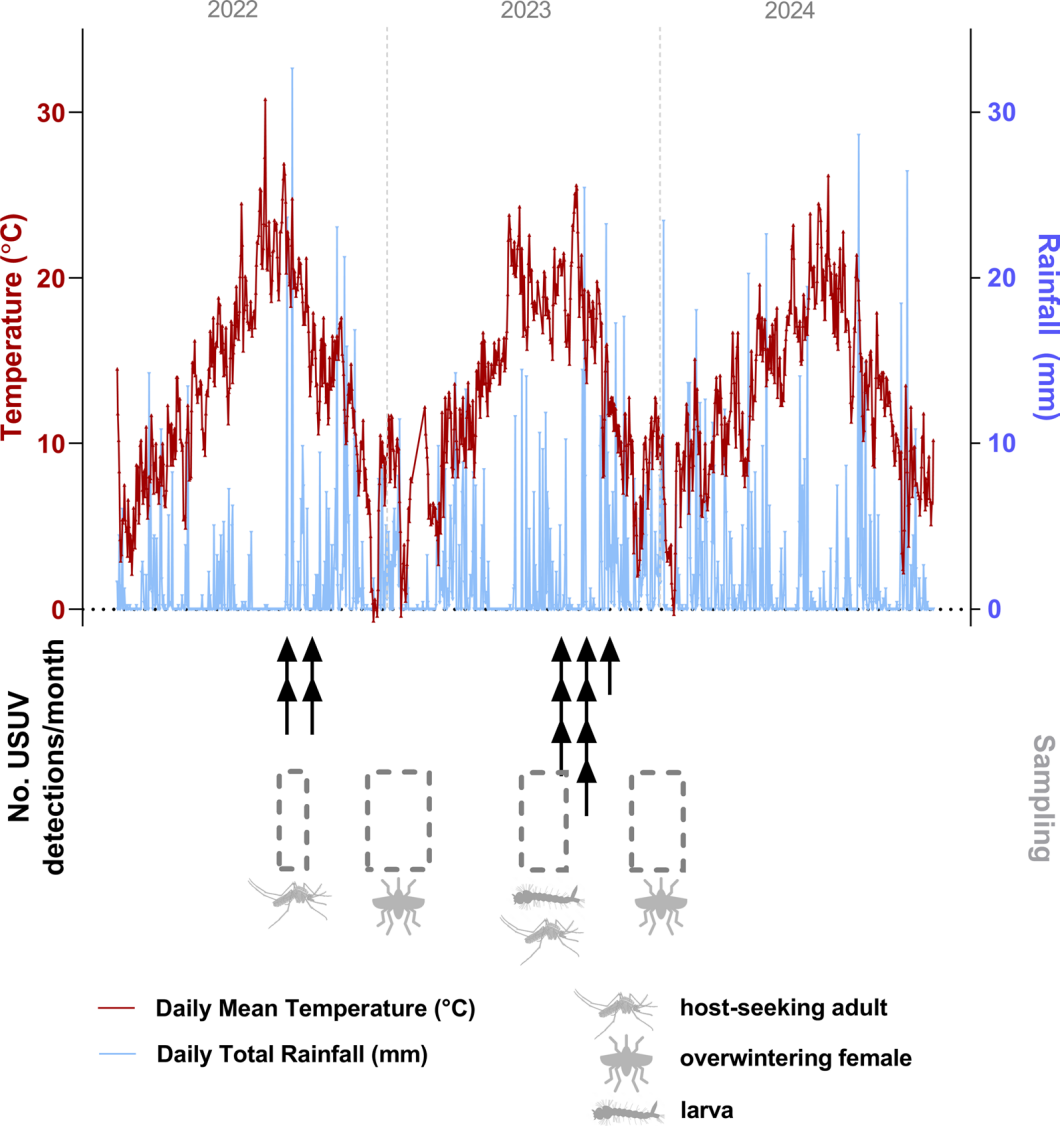
Sampling timepoints capture points in time critical for arbovirus circulation in Greater London. Depicted are the daily mean temperature and daily total rainfall for St James’s Park, London (0900–0900 hours; Met Office National Meteorological Archive) for 2022–2024. Black arrows indicate the number of confirmed cases of Usutu virus (*USUV*) detected in birds per month as an indicator of arbovirus circulation in Greater London.* Grey boxes* indicate the sampling periods as well as the type of mosquito (host-seeking, overwintering, larvae) collected

A comparison of our chosen sampling dates with weather data for 2022 to 2024 provided by the Met Office National Meteorological Archive, as well as confirmed USUV detections in Greater London as an indicator of arbovirus circulation, highlighted the optimal set-up for our sampling strategy to detect circulating arboviruses in a local mosquito population (Fig. 2). Collection of host-seeking mosquitoes in September 2022 followed a period of very hot and dry weather (August 2022; average daily mean temperature, 21.79 ºC, average daily total rainfall, 2.12 mm), coinciding with the detection of USUV in a total of four birds (in preparation) in Greater London. In summer 2023 larvae were collected during a period of hot and dry weather (July 2023; average daily mean temperature, 19.16 ºC, average daily total rainfall, 2.58 mm), which was followed by the detection of a total of eight USUV- positive birds (in preparation) in Greater London between August and October 2023. These data confirm the likelihood of prevalent arboviruses being transmitted by host-seeking adults during these time periods at the sampling site, which were likely detectable by PCR.

All extracted RNA samples from mosquito pools were tested for SINV and BATV by using RT-PCR. No amplification of virus-specific sequences was observed in any of the samples, indicating that there was no evidence for the presence of SINV and BATV genomes in the mosquito populations tested that covered three different stages (Table 1).

**Table 1:** Summary of mosquito samples tested for Sindbis virus and Batai virus

Month	Total no. mosquitoes tested	Sindbis virus positive	Batai virus positive
September 2022	156	0	0
November 2022	33	0	0
December 2022	43	0	0
January 2023	221	0	0
February 2023	143	0	0
June 2023	139	0	0
July 2023	818	0	0
August 2023	5330	0	0
September 2023	350	0	0
November 2023	484	0	0
December 2023	481	0	0
January 2023	279	0	0
Total	8477	0	0

No positive samples were detected across any of the months

## Discussion

SINV and BATV are both globally distributed arboviruses with frequent detections in mainland Europe, and thus pose a potential threat to animal and human health. However, their presence and activity in the UK remain poorly investigated. The findings of this study suggest that there was no circulation of SINV or BATV in three different mosquito stages at the sentinel location used in the present study in southern England between 2022 and 2024. Based on our sample size of host-seeking females (*n* = 5496), our approach would have enabled us to detect SINV and BATV at a minimum observed prevalence of 0.0182%. Published data from Sweden, where SINV is endemic, suggest that this would have enabled the detection of SINV in an outbreak (reported infection rates of 10.0/1000 in 2002) and in a non-outbreak year in Sweden (reported infection rate of 4.9/1000 in 2001) [40]. Our results align with the lack of documented cases of SINV and BATV infection in the UK and their absence in a similar study conducted at Chester Zoo in northeast England [18]. However, future studies that also collect molecular and serological data from a number of different birds and mammals housed at London Zoo would complement mosquito surveillance studies and provide additional information about past or present SINV and BATV incursions [31].

Data for other arboviruses in Europe suggest that the circulation of vector-borne viruses increases in hot, dry summers [41, 42]. Although our samples, which were collected during the hottest and driest periods of the summers of 2022 and 2023, did not suggest the presence of SINV or BATV, active surveillance during specific weather conditions will increase the chance of detecting their emergence. Local SINV circulation was reported from the Netherlands for our sampling period [43], and in Spain SINV was detected for the first time in 2022, with data suggesting an introduction there in 2017 [44].

Recently published surveillance data of USUV in mosquitoes conducted at London Zoo between 2022 and 2024 reported the detection of USUV RNA in host-seeking adults as well as in one pool of mosquitoes reared from collected larvae [32], highlighting that the sampling time points chosen in the present study did indeed capture critical time points of arbovirus circulation in Greater London. The data underline both the presence of circulating arboviruses at the chosen sentinel location and time, as well as the relevance of studying the contribution of vertical transmission to the overall persistence of arboviruses in a specific location.

Additionally, the absence of SINV and BATV detections at London Zoo does not rule out their potential presence in other parts of the country, as indicated by the detection of seroconversion in sentinel chickens in Cambridgeshire in 2004 [17]. Studies on BATV in Europe highlight that, despite its established presence, the virus is only infrequently detected across mosquitoes, mammals, birds, and humans (reviewed in [8]). This suggests that both the prevalence and viral titres in vector populations might be inherently low, aligning with the challenges of detecting BATV in ecological and epidemiological studies. As recommended previously, collecting serological data from sentinel locations such as a zoo could add sensitivity to studies investigating arbovirus prevalence. A limiting factor would then, however, be the occurrence of cross-reactive antibodies produced as a response to the presence of viruses belonging to the same serogroup [4, 45, 46], requiring more specific virus neutralisation tests (as, for example, performed in [47] to distinguish USUV and WNV). In the case of assessing SINV and BATV prevalence, this is complicated by the fact that potentially co-circulating alphaviruses and orthobunyaviruses are highly understudied in Europe and little is known about their prevalence in the UK [46, 48].

Overall, more active surveillance data from other areas of southeast England is needed. In addition, acquiring data on potential hosts to corroborate these findings is highly recommended, alongside the development of diagnostic tests to distinguish arboviruses belonging to the same serogroup.

Early detection of viral activity within vector populations would significantly enhance the ability to pinpoint high-risk areas to target public and animal health messaging. Additionally, guided vector control strategies would prevent viral spread. Continuous monitoring would additionally provide insights into how environmental and climatic changes influence virus activity and mosquito behaviour. This information could then support the development of predictive models, which would strengthen preparedness and response strategies against future arboviral threats.

## Conclusions

Our findings indicate that SINV and BATV were not actively circulating in the sampled area during the study, which provides valuable baseline data for arbovirus surveillance in the UK. The presence of diverse bird populations at London Zoo highlights the potential for sentinel surveillance to detect the early signs of viral emergence. Future research should expand molecular and serological monitoring across additional UK sites to strengthen preparedness for potential SINV and BATV emergence.

## Supplementary Information

Supplementary material 1: Table 1. Overview of traps and locations organised by collection type.

## Data Availability

Data supporting the main conclusions of this study are included in the manuscript.

## Abbreviations

BATV: Batai virus
qPCR: Quantitative Polymerase chain reaction
RNA: Ribonucleic acid
RT-qPCR: Real time–quantitative Polymerase chain reaction
SINV: Sindbis virus
USUV: Usutu virus
WNV: West Nile virus
