# Supplementary material for: Enhanced surveillance to assess the presence of Sindbis and Batai virus in mosquito populations at an urban zoo in the United Kingdom
Source: Parasit Vectors. 2025 Dec 7;19:25. doi: 10.1186/s13071-025-07149-4 (PMC12797647; doi:10.1186/s13071-025-07149-4)
Supplement: Supplementary file 1 — Supplementary material 1: Table 1. Overview of traps and locations organised by collection type. [file 13071_2025_7149_MOESM1_ESM.docx]

| **Type collected** | **Location** | **No. of traps** | **Trap type** | **What3Word** |
| --- | --- | --- | --- | --- |
| Host-seeking females | African (‘Safari’) Aviary (external) | 2 | Biogents trap | custom.eggs.trip, before.cope.cliff |
|  | African (‘Safari’) Aviary (external) |  | Resting collection | perky.heats.deeper |
|  | Mappins service area (internal) | 1 | Biogents mosquitaire trap | curiosity.lonely.tennis |
|  | Mappins service area (internal) | 1 | Biogents sentinel trap | scores.deeply.opera, |
|  | Mappins service area (internal) |  | Resting collection | agenda.score.drives |
|  | Penguins service area (external) | 1 | Biogents sentinel trap | crown.lend.gloves |
|  | Penguins service area (external) | 2 | Biogents mosquitaire trap | universally.easy.raves |
|  |  |  |  | jump.gates.ballots |
|  | Tiger Territory (external) |  | Resting collection | intend.plank.pocket |
|  | Vet Hospital service area (external) |  | Magnet trap | paper.glee.office |
| Overwintering females | Colobus monkey pump room (internal) |  | Resting collection | rescue.career.tubes |
|  | Mappins service area (internal) |  | Resting collection | popped.usual.spot |
| Larvae | African (‘Safari’) Aviary (external) | 3 | Open water pools | flock.secret.files |
|  | Blackburn Building 1 (external) | 1 | Open water pools | gent.pulled.sobs |
|  | Blackburn Building 2 (external) | 1 | Open water pools | gravy.riots.bugs |
|  | Mappins service area (external) | 3 | Open water pools | formal.blur.raves |
|  |  |  |  | marked.stroke.pound |
|  |  |  |  | about.filed.rivers |
|  | Northbank (external) | 2 | Open water pools | garden.fairly.hero |
|  | Penguins service area (external) | 3 | Open water pools | common.swear.items |
|  |  |  |  | total.repay.admiral |
|  |  |  |  | desks.darker. oval |
|  | Vet Hospital service area (external) | 2 | Open water pools | birds.zoom.divide |
|  |  |  |  | issued.regime.cable |
